# Editorial: Lighter and more efficient robotic joints in prostheses and exoskeletons: Design, actuation and control

**DOI:** 10.3389/frobt.2023.1063712

**Published:** 2023-03-07

**Authors:** Yuanxi Sun, Long Bai, Dianbiao Dong

**Affiliations:** ^1^ State Key Laboratory of Mechanical Transmission, Chongqing University, Chongqing, China; ^2^ School of Mechanical Engineering, Northwestern Polytechnical University, Xi’an, China

**Keywords:** robotic joint, prosthesis, exoskeleton, variable stiffness actuator, control

The robot joint is the foundation of prosthetics and exoskeletons, which is the key to realizing human-computer interaction for diverse motion characteristics. From the perspective of physiological anatomy, human joints show unique structural characteristics. For example, the knee joint provides a compound joint rotation formed by sliding and rotating between the femur and the tibia, thus realizing a unique physiological characteristic of the Instantaneous Center of Rotation (ICR). Therefore, simply using a uniaxial hinge as a knee prosthesis or exoskeleton may cause uncoordinated movement between external devices and the human body. Certainly, these unique joint characteristics can be improved by using multi-bar mechanisms or some combined mechanisms, leading to matched and imitated physiological performance of human joints from the perspective of bionics (Sun et al., 2021). However, it should be noted that the weight and size of these complex mechanism-based joints will increase, so how to balance and make the trade-off between these design points to achieve lightweight robotic joints remains of further investigation.

From the perspective of actuation, the human body retains a compliant actuating system composed of muscles and bones, which have excellent task adaptability under various movement conditions. Therefore, if a robotic joint is actuated only by a motor-reducer system, it may cause problems such as higher energy consumption and less impact resistance, resulting in compromised adaptation to the complex movement conditions of prosthetics and exoskeletons. Taking an ankle-foot prosthesis or exoskeleton as an example, when the subject suddenly stops or steps in the air for some reason, the large instantaneous force on the sole will be directly reflected on the motor, causing the motor to stall or even burn out. However, in human joints, external shocks and load fluctuations have less impact on the soft muscles. Moreover, this external mechanical energy may also be absorbed and released at the right time and location. Inspired by this phenomenon, compliant actuators such as variable stiffness actuators (Wolf et al., 2015) and some functional material-based artificial muscles (Mirvakili and Hunter, 2018) have emerged. However, designing these compliant actuators to meet the specific dynamics conditions of the corresponding robotic joint is still challenging.

From the control point of robotic joints, the essence is to make the prosthesis and exoskeleton keep up with human motion intentions. This often requires detailed joint control systems modeling or a supervised or unsupervised data-driven approach to collect large amounts of data for model training. Moreover, accurate joint motion detection and human intention recognition are essential for efficient joint control due to the need for closed-loop control. Typically, inertial sensors, load cells, sEMG sensors, EEG sensors, visual approach, and physiological indicators (e.g., metabolism, blood oxygen, heartbeat, etc.) can be used to percept human motion intentions (Sun et al., 2022). However, individual differences, physiology, gender, age, weight, and life patterns, will all impact the human intent recognition effect. This requires the recognition model to have excellent generalization ability to adapt to various subjects.

After the peer review process, this Research Topic accepted four research articles. Pan et al. proposed a novel robot-aided upper limb rehabilitation system *via* visual, auditory, and tactile feedback in combination with a game-based training task. Compared with only audio-visual-based rehabilitation approaches, the design in this article can help patients enjoy training, improving their motivation and active participation in the tasks. Assfaw and Seid designed a higher-order sliding mode controller for an MR damper-based semi-active prosthetic knee. The authors first built the dynamics model of the single-axis knee joint based on Lagrangian mechanics and then analyzed the MRF using the Bingham Plastic model. The proposed super twisting sliding mode controller is verified in MATLAB simulations, and the results indicate that the knee profile tracking accuracy is improved and the walking gait is near the natural gait. Bai et al. proposed a bio-inspired hydraulic actuator that could imitate the configuration of the human muscle *via* a single power source and multiple plunger pistons. Each piston chamber is connected to a two-position three-way switch valve, based on which the actuator can output different forces by recruiting the effective areas of plunger pistons in a fixed order. In addition, a robotic arm was designed and used to demonstrate that the proposed actuator can improve the efficiency of the hydraulic system with variable loads. Ramos et al. compared the mechanical properties of hand exoskeleton textile and silicone actuators in terms of physical dimensions, the air pressure required to achieve a full bending motion, and the forces generated at the tip of the actuator. The results indicate that actuators constructed using pleated textile techniques have greater potential in terms of 78% lighter weight, 72% less actuation pressure requirement, 48% more bending force, and 35% more block force for the construction of hand exoskeleton.

In summary, all the articles presented on this Research Topic have provided novel and in-depth understandings of the design, actuation, and control of the prosthesis and exoskeleton, facilitating readers to get a comprehensive understanding and cognition of the current development direction and application prospects of robotic joints.

## References

[B1] Mirvakili S. M. Hunter I. W. (2018). Artificial muscles: Mechanisms, applications, and challenges. Adv. Mater. 30, 1704407. 10.1002/adma.201704407 29250838

[B2] Sun Y. Tang H. Tang Y. Zheng J. Dong D. Chen X. (2021). Review of recent progress in robotic knee prosthesis related techniques: Structure, actuation and control. J. Bionic Eng. 18, 764–785. 10.1007/s42235-021-0065-4

[B3] Sun Y. Tang Y. Zheng J. Dong D. Chen X. Bai L. (2022). From sensing to control of lower limb exoskeleton: A systematic review. Annu. Rev. Control 53, 83–96. 10.1016/j.arcontrol.2022.04.003

[B4] Wolf S. Grioli G. Eiberger O. Friedl W. Grebenstein M. Höppner H. (2015). Variable stiffness actuators: Review on design and components. IEEE/ASME Trans. mechatronics 21, 2418–2430. 10.1109/tmech.2015.2501019

